# Supplementary material for: Mendelian randomization identifies circulating proteins as biomarkers for age at menarche and age at natural menopause
Source: Commun Biol. 2024 Jan 6;7:47. doi: 10.1038/s42003-023-05737-7 (PMC10771430; doi:10.1038/s42003-023-05737-7)
Supplement: Supplementary file 2 — Description of Additional Supplementary Files [file 42003_2023_5737_MOESM2_ESM.pdf]

Description of Additional Supplementary Files

File name: Supplementary Data 1
Description: Information on genotyping, proteomic platform and imputation panel for the six proteomic GWAS and the GWAS for AAM and ANM.

File name: Supplementary Data 2
Description: MR power calculation for AAM. Proteins with less than 80% power are highlighted.

File name: Supplementary Data 3
Description: MR power calculation for ANM. Proteins with less than 80% power are highlighted.

File name: Supplementary Data 4
Description: Two sample MR results for AAM using directly matched sentinel cis-pQTLs. Bold letters highlight the Bonferroni correction cut-off and grey font is used for cross-validated proteins from multiple proteomic GWAS.

File name: Supplementary Data 5
Description: Strength of the genetic instruments for AAM.

File name: Supplementary Data 6
Description: Colocalization analyses for MR-prioritized proteins and AAM results for four hypotheses (H) with orange highlighting H3 , and blue highlighting H4 as hypotheses with the maximum posterior probabilities.

File name: Supplementary Data 7
Description: Colocalization with SuSiE (B) analyses for MR-prioritized proteins and AAM results for four hypotheses (H) with orange highlighting H3 , and blue highlighting H4 as hypotheses with the maximum posterior probabilities.

File name: Supplementary Data 8
Description: Strength of the genetic instruments for ANM.

File name: Supplementary Data 9
Description: Two sample MR results for ANM using directly matched sentinel cis-pQTLs. Bold letters highlight the Bonferroni correction cut-off and grey font is used for cross-validated proteins from multiple proteomic GWAS.

File name: Supplementary Data 10
Description: Colocalization analyses for MR-prioritized proteins and ANM results for four hypotheses (H) with orange highlighting H3 , and blue highlighting H4 as hypotheses with the maximum posterior probabilities.

File name: Supplementary Data 11
Description: Colocalization with SuSiE analyses for MR-prioritized proteins and ANM results for four hypotheses (H) with orange highlighting H3 , and blue highlighting H4 as hypotheses with the maximum posterior probabilities.

File name: Supplementary Data 12
Description: HyperColoc analysis with GCKR, AAM and ANM.

File name: Supplementary Data 13
Description: Steiger Test results with protein levels as exposure and AAM as outcome. Yellow highlight designates p-values lower than 0.05 and red highligt designates p-values lower than a Bonferroni-corrected pvalue of 0.0025.

File name: Supplementary Data 14
Description: Steiger Test results with protein levels as exposure and ANM as outcome. Yellow highlight designates p-values lower than 0.05 and red highligt designates p-values lower than a Bonferroni-corrected pvalue of 0.0025.

File name: Supplementary Data 15
Description: Reverse MR results with AAM as exposure and protein levels as outcome. Yellow highlight designates p-values lower than 0.05 and red highligt designates p-values lower than a Bonferroni-corrected p value of 0.0025.

File name: Supplementary Data 16
Description: Reverse MR results with ANM as exposure and protein levels as outcome. Yellow highlight designates p-values lower than 0.05.

File name: Supplementary Data 17
Description: Phenoscanner search for GWAS associations of cis-pQTLs of the MR-prioritized proteins for AAM with complex traits. Anthropometric traits are denoted in bold. Associations with age at menarche or age at menopause are highlighted in red.

File name: Supplementary Data 18
Description: Phenoscanner search for GWAS associations of cis-pQTLs of the MR-prioritized proteins for ANM with complex traits. Anthropometric traits are denoted in bold. Associations with age at menarche or age at menopause are highlighted in red.

File name: Supplementary Data 19
Description: Two-step network MR using colocalization-prioritized proteins, childhood BMI and AAM. Proteins with significant indirect effects are highlighted.

File name: Supplementary Data 20
Description: Two-step network MR using colocalization-prioritized proteins, adult BMI and ANM. Proteins with significant indirect effects are highlighted.

File name: Supplementary Data 21
Description: MVMR using colocalization-prioritized proteins, childhood BMI and AAM. Significant effects are highlighted.

File name: Supplementary Data 22
Description:

File name: Supplementary Data 23
Description: MVMR using colocalization-prioritized proteins, adult BMI and ANM. Significant effects are highlighted.

File name: Supplementary Data 24
Description: Assesment of association of cis-pQTLs for the candidate proteins with nearby protein altering variants (PAV) and ANM.

File name: Supplementary Data 25
Description: Results for 13 colocalization-prioritized proteins on AAM using GeneMANIA for physical interaction (1), function (2), and involving pathway (3).

File name: Supplementary Data 26
Description: Annotation analysis using Metascape for colocalization-prioritized proteins for AAM with colors gradient p-value.

File name: Supplementary Data 27
Description: Results of gene set enrichment analysis (GTEx v8) using FUMA for colocalization-prioritized proteins for AAM.

File name: Supplementary Data 28
Description: Results for 7 colocalization-prioritized proteins on ANM using GeneMANIA for physical interaction (1), function (2), and involving pathway (3).

File name: Supplementary Data 29
Description: Annotation analysis using Metascape for colocalization-prioritized proteins for ANM with colors gradient p-value.

File name: Supplementary Data 30
Description: Results of gene set enrichment analysis (GTEx v8) using FUMA for colocalization-prioritized proteins for ANM.

File name: Supplementary Data 31
Description: Drug target search for MR-prioritized proteins for AAM (source: OpenTargets).

File name: Supplementary Data 32
Description: Drugs target search for MR-prioritized proteins for ANM (source: OpenTargets).
