# Supplementary material for: Mendelian randomization identifies circulating proteins as biomarkers for age at menarche and age at natural menopause
Source: Commun Biol. 2024 Jan 6;7:47. doi: 10.1038/s42003-023-05737-7 (PMC10771430; doi:10.1038/s42003-023-05737-7)
Supplement: Supplementary file 4 — Reporting Summary [file 42003_2023_5737_MOESM4_ESM.pdf]

nature portfolio

Corresponding author(s): Despoina Manousaki

Last updated by author(s): Dec 20, 2023

Reporting Summary

Nature Portfolio wishes to improve the reproducibility of the work that we publish. This form provides structure for consistency and transparency in reporting. For further information on Nature Portfolio policies, see our Editorial Policies and the Editorial Policy Checklist.

Statistics

For all statistical analyses, confirm that the following items are present in the figure legend, table legend, main text, or Methods section.

n/a	Confirmed	
☐	☒	The exact sample size (*n*) for each experimental group/condition, given as a discrete number and unit of measurement
☐	☒	A statement on whether measurements were taken from distinct samples or whether the same sample was measured repeatedly
☐	☒	The statistical test(s) used AND whether they are one- or two-sided<br>*Only common tests should be described solely by name; describe more complex techniques in the Methods section.*
☐	☒	A description of all covariates tested
☐	☒	A description of any assumptions or corrections, such as tests of normality and adjustment for multiple comparisons
☐	☒	A full description of the statistical parameters including central tendency (e.g. means) or other basic estimates (e.g. regression coefficient) AND variation (e.g. standard deviation) or associated estimates of uncertainty (e.g. confidence intervals)
☐	☒	For null hypothesis testing, the test statistic (e.g. *F*, *t*, *r*) with confidence intervals, effect sizes, degrees of freedom and *P* value noted<br>*Give P values as exact values whenever suitable.*
☒	☐	For Bayesian analysis, information on the choice of priors and Markov chain Monte Carlo settings
☒	☐	For hierarchical and complex designs, identification of the appropriate level for tests and full reporting of outcomes
☐	☒	Estimates of effect sizes (e.g. Cohen's *d*, Pearson's *r*), indicating how they were calculated

Our web collection on statistics for biologists contains articles on many of the points above.

Software and code

Policy information about availability of computer code

Data collection	Not applicable
Data analysis	All R codes used to generate the results of the MR and colocalization analyses are available on github or Zenedo.

For manuscripts utilizing custom algorithms or software that are central to the research but not yet described in published literature, software must be made available to editors and reviewers. We strongly encourage code deposition in a community repository (e.g. GitHub). See the Nature Portfolio guidelines for submitting code & software for further information.

Data

Policy information about availability of data

All manuscripts must include a data availability statement. This statement should provide the following information, where applicable:

- Accession codes, unique identifiers, or web links for publicly available datasets
- A description of any restrictions on data availability
- For clinical datasets or third party data, please ensure that the statement adheres to our policy

Data on circulating proteins from seven GWAS were obtained from the following sources and see Supplementary Data 1 for accesion number :
Ferkingstad, E. et al https://download.decode.is/form/folder/proteomics,
Sun, B.B. et al.UKBB https://static-content.springer.com/esm/art%3A10.1038%2Fs41586-022-04394-w/MediaObjects/41586_2022_4394_MOESM4_ESM.xlsx

Sun, B.B. et al. https://www.ebi.ac.uk/gwas/publications/29875488
Folkersen, L. et al. https://www.ebi.ac.uk/gwas/publications/33067605
Yao, C. et al. https://www.ebi.ac.uk/gwas/publications/30111768
Suhre, K. et al. https://www.ebi.ac.uk/gwas/publications/28240269
Emilsson, V. et al. https://www.ebi.ac.uk/gwas/publications/30072576
Vogelezang, S et al. https://www.ebi.ac.uk/gwas/publications/33045005 Yengo, L et al. https://www.ebi.ac.uk/gwas/publications/30124842
The data on the ages at menarche and natural menopause were obtained from the REPROGEN Consortium GWAS. https://www.reprogen.org/data_download.html

Research involving human participants, their data, or biological material

Policy information about studies with human participants or human data. See also policy information about sex, gender (identity/presentation), and sexual orientation and race, ethnicity and racism.

Reporting on sex and gender	All participants in AAM and ANM GWAS were females.<br>Participants in all proteomics and BMI GWAS were of both sexes.<br>Gender was not taken into consideration in the analyses.
Reporting on race, ethnicity, or other socially relevant groupings	All GWAS studies used in this analysis were of European descent.
Population characteristics	Please refer to Supplemental Table 1 of the manuscript for description of the GWAS populations
Recruitment	Not applicable
Ethics oversight	All participants in the GWAS used for this study provided informed consent.

Note that full information on the approval of the study protocol must also be provided in the manuscript.

Field-specific reporting

Please select the one below that is the best fit for your research. If you are not sure, read the appropriate sections before making your selection.

☒ Life sciences ☐ Behavioural & social sciences ☐ Ecological, evolutionary & environmental sciences

For a reference copy of the document with all sections, see nature.com/documents/nr-reporting-summary-flat.pdf

Life sciences study design

All studies must disclose on these points even when the disclosure is negative.

Sample size	Please refer to Supplemental Table 1 for sample sizes of all GWAS sources used in this study
Data exclusions	not applicable
Replication	not applicable
Randomization	not applicable
Blinding	not applicable

Reporting for specific materials, systems and methods

We require information from authors about some types of materials, experimental systems and methods used in many studies. Here, indicate whether each material, system or method listed is relevant to your study. If you are not sure if a list item applies to your research, read the appropriate section before selecting a response.

Materials & experimental systems

n/a	Involved in the study
☒	☐ Antibodies
☒	☐ Eukaryotic cell lines
☒	☐ Palaeontology and archaeology
☒	☐ Animals and other organisms
☒	☐ Clinical data
☒	☐ Dual use research of concern
☒	☐ Plants

Methods

n/a	Involved in the study
☒	☐ ChIP-seq
☒	☐ Flow cytometry
☒	☐ MRI-based neuroimaging

Plants

Seed stocks	not applicable
Novel plant genotypes	not aplicable
Authentication	not applicable
